# Trends in Israeli clinical trials registration “MyTrial”

**DOI:** 10.1186/s13584-024-00643-7

**Published:** 2024-10-24

**Authors:** Anat Engel, Ornit Cohen

**Affiliations:** 1 Health Administration Department Wolfson Medical Center, Holon, Israel; 2 Research and Innovation Authority, Wolfson Medical Center, Holon, Israel

**Keywords:** Clinical trials, Registry, MyTrial, ClinicalTrials.gov, Israel

## Abstract

**Background:**

Clinical trial registration is critical for research transparency and integrity. Since 2005, the Declaration of Helsinki has required prospective registration of trials before subject recruitment. In Israel, the MyTrial registry was established in 2015 to register interventional trials and became mandatory in 2016 for ethical approval. The study aimed to analyze the registration practices, challenges, and trends in clinical trial registration in Israel using the local registry “MyTrial”.

**Methods:**

A total of 3,895 clinical trial records from 2011 to December 2022 were retrieved from the MyTrial platform and subjected to descriptive analysis.

**Results:**

A significant increase occurred from 2016 to 2021 due to mandated registration, with a peak in 2020 (733 trials) and a decrease in 2022 (462 trials), likely due to COVID-19. Most of the trials included drugs (56%) or medical devices (33%). Geographically, 53% were from central Israel. Only 39% of the patients were registered at both MyTrial and ClinicalTrials.gov. 65% had no blinding. 47% featured unregistered products. 56% had not started recruitment. Since 2016, the number of advanced therapy trials has steadily increased, reaching 19 in 2022. There are gaps between registered trials and official government reports.

**Conclusion:**

These findings provide valuable insights into the current landscape of clinical trial registration in Israel and highlight the need for improvements in compliance with prospective registration and adherence to the WHO-ICTRP standards.

## Significance

What’s Known on this Subject

Clinical trial registration has been increasingly recognized as important for research transparency and integrity since requirements were introduced in the 2005 Declaration of Helsinki.

## Introduction

Clinical trial registration trends have shown an increase in the number of trials registered over the years, indicating growing recognition of the importance of transparency and accountability in clinical research [1]. However, achieving full compliance with prospective registration remains challenging, with some trials being retrospectively registered. Since 2005, the 7th Declaration of Helsinki has indicated that ‘Every clinical trial must be registered in a publicly accessible database before recruitment of the first subject’ [2], meaning that before the first participant is recruited, the information on the trial must be captured in a publicly accessible database. The registry promotes transparency and accountability in clinical research by publicly providing information about ongoing and completed trials available [3–5].

To address the need for trial registration, the World Health Organization (WHO) has developed global standards for clinical trial registries (6–7). These standards aim to promote harmonization and quality in clinical trial registries to improve transparency and global access to clinical trial information [8]. Over the years, many countries have established their own local clinical trial registry according to WHO International Clinical Trials Registry Platform (ICTRP) standards [9–11]. Although establishing a public registry for clinical trials may seem beneficial, opposing arguments question its effectiveness and necessity. Critics argue that the registration process can be burdensome for researchers by adding unnecessary administrative tasks. Furthermore, due to resource constraints, trial registration may deter smaller research institutions and independent researchers from conducting trials [12].

Several factors contribute to non-compliance with trial registration, including lack of awareness, delays in registration, incomplete provision of information, and the existence of numerous trial registration databases, such as ClinicalTrials.gov and WHO-ICTRP, which may result in confusion among researchers [13]. These factors hinder the potential benefits of clinical trial registration, such as improving transparency, reducing publication bias, and facilitating meta-analysis [14].

In Israel, the local clinical trials registry named ‘MyTrial’ was established in 2016 [15], although it does not adhere completely to the WHO-ICTRP standards. MyTrial is a repository of information on clinical studies conducted in Israel and is available in Hebrew and English. Since its establishment and support by the MOH guidelines, each interventional clinical trial in Israel must have a registered number on MyTrial.Gov before IRB approval.

This study provides valuable insights into the registration practices, challenges, and trends in clinical trial registration in Israel. By conducting a comprehensive analysis, this study aims to deepen the understanding of the current landscape of clinical trial registration and provide regulatory authorities with recommendations for improvement.

## Methods

The dataset was obtained from the MyTrial website (https://my.health.gov.il/CliniTrials/Pages/Home.aspx) in XML format. It consists of various variables, including registration status, type of study, disease condition, demographics of trial participants (e.g., gender), information about the sponsor, type of intervention used, funding source, trial design’s intervention model utilized, phase of the trial, and overall recruitment status.

As of December 31, 2022, MyTrial had 3896 prospective trials registered. Upon registration, all trials are marked based on their status, and this marking is unchangeable after registration. The analysis involved national evaluation of all interventional clinical studies, irrespective of their stage or outcome. A comparison was made between the trial numbers, phases, classifications, and medical interventions to tackle the challenges and key issues identified within “MyTrial”.

As the MOH officially mandated in 2016 that interventional trials must be registered with each ethics committee beforehand, the initial data entry in 2016 included studies that had already been initiated.

We conducted a descriptive analysis of the use and trends of registered trials in MyTrial to understand the pattern of trial registration over the years using SPSS v.27. Statistical significance was set at *P* < 0.05. Patients and/or the public were not involved in this research’s design, conduct, reporting, or dissemination.

## Results

**Fig. 1:**
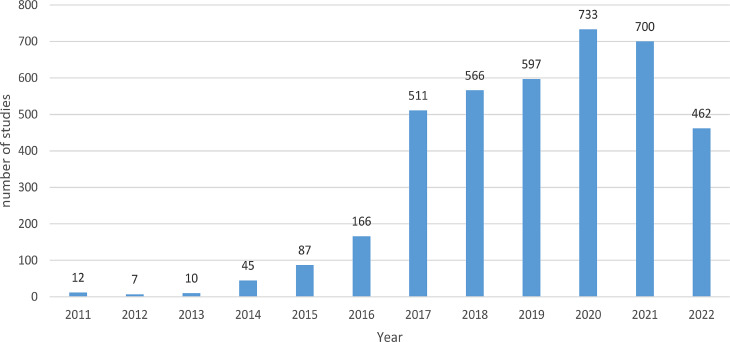
Trials registration trend in the Clinical Trials Registry of Israel. Number of retrospective and prospective registrations on MyTrial by year. We conducted a descriptive analysis of the trials registered in MyTrial and showed the number of trials registered per year on the x-axis. The y-axis represents the number of trials out of the Total

A total of 3895 sources of clinical trial records were retrieved from the MyTrial platform website and used for our analysis. The Israeli IRB approved All of these procedures, starting from 2011 to December 2022. MyTrial platform has grown substantially since it was first launched, with each year showing a steady increase in the number of trials registered and in the study type. Figure 1 shows the significant increase in the number of interventional trials performed from 2011 to 2022 in Israel. Since 2016, MyTrial has registered a unique MOH number that is mandatory for ethical approval; therefore, the number of trials has been gradually increasing. Each researcher was required to indicate the type of intervention for each trial on the MyTrial platform. The majority of trials fell under the “Drug” category (56%), followed by “Medical Device” (33%). Most trials were industry-sponsored and initiated (56%; 2187/3895), while investigator-initiated trials accounted for 44% (1709/3895). Regarding the geographic distribution within Israel, a significant proportion of trials (53%) occurred in and around the central region. Other regions where studies have been conducted include the north (22%), Tel Aviv (13%), Jerusalem (8%), southern region (4%), and Ashkelon (1%) (Table 1). Oncology trials constituted the largest proportion of clinical trials in Israel, accounting for 17% of all trials. This was followed by the Neurology and Cardiology categories, which represented approximately 7% of the total trials (Table 2). In terms of registration, 39% of the trials were registered on both the MyTrial and NIH registry platforms. The methods used for blinding varied significantly between these trials. Approximately 65% of the studies were conducted without any blinding procedures, while approximately 30% utilized double-blind techniques. Only 5% of the trials employed a single-blind approach. Moreover, the product registration status varied in these trials, with 47% featuring unregistered products, 34% involving products registered both in Israel and internationally, 11% exclusively in Israel, and 7% solely internationally. Concerning recruitment status, 56% of the studies did not initiate recruitment, whereas 26% were actively recruiting participants. The remaining participants either concluded (10%) or completed recruitment (8%).

**Table 1:** Registration data set of the clinical trials Registry of Israel

Variable	Category	*n*	%
Study Category	Drug	2152	56%
	Medical Device	1265	33%
	No product	351	9%
	Therapies	100	3%
	Genetic	1	0%
Study Location in Israel	CENTER	2051	53%
	NORTH	857	22%
	TLV	498	13%
	JERUSALEM	297	8%
	SOUTH	144	4%
	ASHKELON	46	1%
NIH Register	No	2364	61%
	Yes	1532	39%
Randomization	No	1917	49%
	Yes	1979	51%
Blindness	No	2523	65%
	Double-blind	1161	30%
	Single blind	212	5%
Registered Product	Not registered	1843	47%
	Israel + International	1341	34%
	Israel	426	11%
	International	286	7%
Recruitment Status	Recruitment not begun	2174	56%
	Recruit	998	26%
	End Study	398	10%
	End Recruit	324	8%
Approval year	2011	12	0%
	2012	7	0%
	2013	10	0%
	2014	45	1%
	2015	87	2%
	2016	166	4%
	2017	511	13%
	2018	566	15%
	2019	597	15%
	2020	733	19%
	2021	700	18%
	2022	462	12%
Total		3896	100%

Out of the 3,896 studies analyzed, the majority belong to the “Drug” category (56%), followed by “Medical Device” (33%). There are also studies categorized as “No product” (9%), “Therapies” (3%), and no studies classified as “Genetic”. In terms of geographical distribution within Israel, a significant portion of the studies (53%) are concentrated in the central region. Other regions that have conducted studies include the north (22%), Tel Aviv area (13%), Jerusalem (8%), southern region (4%) and Ashkelon (1%)

Regarding registration, 39% of these studies are registered both in MyTrial and the NIH registry platform. Blinding methods were found to vary across these trials - around 65% were conducted without any blinding procedures in place; however, approximately 30% used double-blind techniques, and 5% employed single-blind approaches

Product registration status varies as well, with 47% featuring unregistered products, 34% of the trials involving products registered both in Israel and internationally, 11% exclusively in Israel, and 7% solely internationally. Recruitment statuses indicate that 56% of studies have not yet initiated recruitment, while 26% are actively recruiting participants. The rest, either have concluded (10%) or completed recruitment (8%)

There is an increasing trend in the distribution of study approval years from 2015 onwards. The highest proportions are seen in 2020 (19%) and 2021 (18%). In contrast, the years 2011 to 2014 each have no studies accounted for

**Table 2:** Disease conditions investigated in the MyTrial platform

	*n*	%
Oncology	657	17%
Neurology	281	7%
Cardiology	260	7%
Hematology	252	6%
OBG	249	6%
Gastro	234	6%
Infection	169	4%
Ophthalmology	168	4%
Endocrinology	149	4%
Psychiatry	131	3%
Lung	126	3%
Dermatology	102	3%
Rheumatology	89	2%
Orthopedic	76	2%
Pediatric	65	2%
Imaging	61	2%
Nephrology	59	2%
Otolaryngology	57	1%
Urology	56	1%
Allergy	54	1%
Hepatology	51	1%
Surgery	51	1%
Rehabilitation	48	1%
Internal	44	1%
Anesthesia	43	1%
Plastic surgery	42	1%
General Medicine	39	1%
Geriatric	38	1%
ICU	35	1%
Dental	34	1%
Psychology	28	1%
Pain	27	1%
Genetic	26	1%
Neurosurgery	25	1%
IVF	21	1%
ER	20	1%
Sleep lab	14	0%
Vascular surgery	10	0%
Palliative	5	0%
Total	3896	100%

**Fig. 2:**
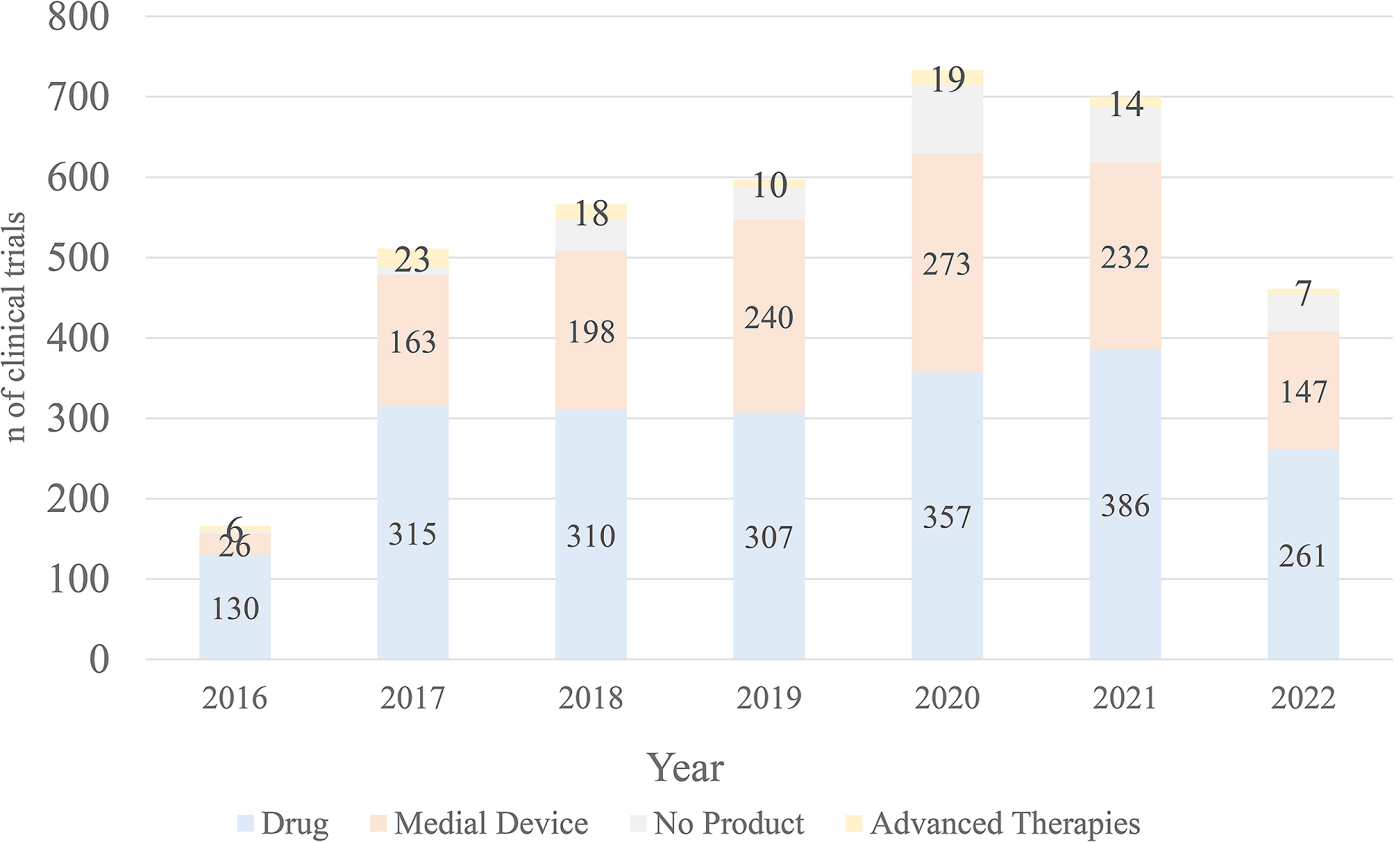
Subtyping the n of studies by their type of intervention per year. Subtyping the number of studies by their type per year, it appeared that the most common trials were medical device trials. The average indicates that every year, 295 ± 76 of the studies in the MyTrial Platform are new investigational drugs, 182 ± 75 are medical devices, 13 ± 6 are advanced therapies, and 42 ± 25 are no investigational products

From 2016 onward, there has been a noticeable trend of consistent growth in clinical trials for Advanced Therapies and Medical Devices, including innovative treatments such as gene therapy, cell therapy, and regenerative medicine. When categorizing the percentage of studies by the type of intervention per year, it appeared that the most common trials were medical device trials. The average indicates that 295 ± 76 of the studies in the MyTrial Platform are new investigational drugs, 182 ± 75 are medical devices, 13 ± 6 are advanced therapies, and 42 ± 25 are no investigational products. There has been a consistent increase in clinical trials involving pharmaceuticals, from 130 in 2016 to 261 in 2022. This suggests that pharmaceutical research remains a significant focus within the scope of this study as other categories experience growth. Clinical trials associated with medical devices have shown some fluctuations, peaking at 273 clinical trials in 2020 (Fig. 2).

## Discussion

Our analysis revealed several trends and challenges in the Israeli Clinical Trial Registry MyTrial. We observed a significant increase in interventional trials registered from 2016 to 2021 due to mandatory guidelines by the Israeli Ministry of Health (MOH) [15], but a decrease in 2022, likely due to the COVID-19 pandemic’s impact on pharmacological research worldwide [16, 17]. There is variation across the number of studies with investigational drugs, medical devices, advanced therapies, and no investigational products.

MyTrial does not demand registering retrospective studies according to the GCP definition [18]; hence, data trials are not in the MyTrial Database, although, according to the Israel MOH report 2021 [21], they are the majority in Israel. While earlier trials focused on pharmaceutical drugs, the Israeli landscape has become more balanced with increased attention paid to medical devices and advanced therapy trials. The rise in advanced therapies aligns with global trends and FDA encouragement [19, 20]. Fluctuations in medical device trials can be influenced by several factors, such as regulatory changes in Medical Device Regulation (MDR) [21] or industry shifts to digital health products.

Although there was partial growth in the registry between 2016 and 2022, a significant gap existed between the official MOH numbers in 2022 [22] and MyTrial, likely due to awareness of the importance of registration and implementation of regulatory policies [23].

Our research suggests that MyTrial does not adhere to the International Standards for Clinical Trial Registries (ICTRP) [24] as they need more crucial information regarding funding, conflicts of interest, recruitment update status, updated recruitment sites, study phases, and results [19]. Furthermore, because of the inability to update existing information on MyTrial as the study status changes, many studies on the platform were incomplete or inaccurate, leading to underreported or inconsistent trial numbers compared with the annual reports from the Ministry of Health (MOH).

It is worth mentioning that the current status of MyTrial and the US clinicaltrials.gov site is such that they do not correspond to each other because of the absence of a specific requirement for the Israeli registry. The Israeli Ministry of Health (MOH) must strive to align with the accuracy of clinicaltrials.gov rather than maintaining a local Hebrew database that is not beneficial for the international needs of investigators. By doing so, the objective of the registry, which is to provide public access to information about all clinical trials involving human beings in Israel, can be achieved effectively.

It is strongly recommended that MyTrial be available in English and Hebrew to enhance the global accessibility of Israeli clinical trial information. A bilingual interface would significantly broaden the registry’s reach, allowing researchers, clinicians, and patients worldwide to access and utilize the valuable data from Israeli clinical trials. This approach aligns with the practices of other successful international registries and reinforces Israel’s position as a key contributor to global clinical research.

Improvement of the incomplete information and update of the status of MyTrial necessitates the integration of trial registration with ethics approval and direct updating from the IT system. This involves linking the Ministry of Health (MOH) with any Institutional Review Board (IRB) approval, simplifying registration processes, ensuring proper registration of all trials in Israel, and minimizing the probability of overlooked or unreported trials. Periodic examination and evaluation of the registration process and study status will facilitate the identification of areas that require enhancement. It is recommended to focus on improving the data validation processes in MyTrial, given that the ClinicalTrials.gov registry has been successful owing to effective validation operating for over 15 years.

To ensure the accuracy of trial information, we strongly suggest implementing data validation measures within MyTrial, following its complete integration with the IRB approval IT system. This will help to reduce discrepancies between MyTrial, ClinicalTrials.gov, and Israeli Ministry of Health (MOH) reports, thereby meeting the requirements of international researchers, sponsors, and healthcare providers.

## Limitation

Israeli regulations require six categories of study classification, yet the registry only encompasses a subset of them. MyTrial varies from both ClinicalTrials.gov and WHO-ICTRP standards [24] to the Israeli annual MOH report [22]. A critical aspect of these standards is the capacity for regular updates to correct missing information, which currently compromises the accuracy of recruitment status. Moreover, the platform interface needs more visibility, making it easier to find new studies.

To address these issues and improve search and update functionalities, the integration of registration via a link with ethics approval and updates is proposed. The assumption is that trial registries should provide comprehensive data covering phase classification, participant numbers, funding sources, and conflicts of interest. Such transparency is crucial for facilitating informed decision-making and recruiting new potential subjects to participate in trials.

## Conclusions

This study examined the progress made by the MyTrial Registry in Israel regarding clinical trial registration. These results indicate that mandatory policies have led to significant advancements in this area, but issues still need to be addressed. The increase in interventional trial registrations between 2016 and 2021 is positive.

However, discrepancies between the MyTrial Registry and official reports underscore the need for improved adherence and enforcement mechanisms. To improve transparency and reduce duplication, it is recommended that trial registration be integrated with institutional ethics approval processes and robust data validation measures be implemented.

Aligning the MyTrial Registry with international standards, particularly those of ClinicalTrials.gov and WHO-ICTRP, is not merely a recommendation but a critical imperative for enhancing the global visibility and utility of Israeli clinical research. This alignment should encompass comprehensive reporting of funding sources, conflicts of interest, current recruitment status, study phases, and results. By adopting these standards, MyTrial can significantly contribute to global research transparency, facilitate international collaborations, and ensure that Israeli clinical trials are fully integrated into the global research ecosystem. It would serve the global research community more effectively and fulfill the registry’s public information mandate.

Future improvements should focus on streamlining the registration processes, ensuring comprehensive trial coverage, and facilitating regular updates. Although this study provides valuable insights into Israel’s clinical trial landscape, further research using complete datasets is necessary to develop targeted strategies to optimize compliance and enhance research integrity. By addressing these challenges, Israel can strengthen its position in the global clinical research arena and contribute to advancing transparent, efficient, and reliable clinical trials.

## Data Availability

Data not available.

## Abbreviations

MOH: Ministry of Health
WHO: World Health Organization
ICTRP: International Clinical Trials Registry Platform
IRB: Institutional Review Board
